# A Novel Method for Sentinel Lymph Node Biopsy by Indocyanine Green Fluorescence Technique in Breast Cancer

**DOI:** 10.3390/cancers2020713

**Published:** 2010-04-27

**Authors:** Tomoharu Sugie, Kassim Abdelazeem Kassim, Megumi Takeuchi, Takashi Hashimoto, Kazuhiko Yamagami, Yoshikazu Masai, Masakazu Toi

**Affiliations:** 1 Department of Breast Surgery, Kyoto University Hospital, 54 Kawara-cho Shogoin, Sakyo-ku Kyoto 606-8507, Japan; E-Mails: megumi817@goo.jp (M.T.); toi@kuhp.kyoto-u.ac.jp (M.T.); 2 Surgical Oncology Department, South Egypt Cancer Institute, Assiut University, El-Methaq St., Mansheit El-Omara square, Assiut, Egypt; E-Mail: kassimabdelazeem@yahoo.com (K.K.); 3 Department of Surgery, Shinko Hospital, 1-4-47, Wakihama-cho, Chuo-ku, Kobe 651-0072, Japan; E-Mails: th095@yahoo.co.jp (T.H.); kazu.yama.-0825@shinkohp.or.jp (K.Y.); 4 Department of Surgery, Kobe City Medical Center General Hospital, 4-6 Minatojima Nakamachi Chuo-ku, Kobe 650-0046, Japan; E-Mail: masai@kcgh.gr.jp (Y.M.)

**Keywords:** sentinel lymph node, indocyanine green (ICG), fluorescence, breast cancer

## Abstract

We investigated the feasibility of sentinel lymph node (SLN) biopsy using indocyanine green (ICG) technique in 411 patients with early breast cancer at three institutes. ICG, a fluorescence source, and blue dye were injected into the subareolar area to enable real-time image-guided surgery and identification of SLN fluorescence after meticulous dissection. The subcutaneous lymphatic channels were precisely detected in all cases. SLN identification rate was 99% (408/411) with a mean of 2.3 nodes identified per patient. Thirty-nine cases (9.5%) had SLNs involved and all of them were ICG positive. Thus, the ICG technique has a high SLN identification rate comparable with that of the radioisotope method.

## 1. Introduction

Sentinel lymph node (SLN) biopsy is now a standard method for evaluating axillary lymph node status in early breast cancer. SLNs are the first point to harbor metastasis in the axilla. They are representative of the actual lymph node status and provide information on the necessity for further management after axillary surgery. If SLNs are not involved, axillary lymph node dissection (ALND) can be avoided, thereby reducing the morbidity associated with surgery. In a randomized trial by Veronesi *et al*., overall five-year survival in the SLN biopsy group was statistically comparable with that of the ALND group. SLN biopsy can negate the need for ALND in patients with negative SLNs [1].

Radioisotopes are currently the standard tracers used to identify SLNs. The combination of a radioisotope and a blue dye enables both a high detection rate and a low false negative rate as noted in ASCO guidelines [2]. However, the use of radioisotopes has disadvantages: their availability is limited at some institutions, the time interval from injection to operation varies, and a special gamma detector device is required for detection. A novel method using an indocyanine green (ICG) fluorescence imaging system has recently been published with reported SLN identification rates of 94–100% [3,4]. In this system, lymphatic flows were traced with a charge-coupled device (CCD), and real-time image guided surgery enabled identification of the correct site for exploring SLNs. Fluorescent lymph nodes were harvested as SLNs, and this technique provided a success rate comparable to that of the radioisotope method in preliminary studies. Here we detail the results of our ICG fluorescence navigation technique for SLN biopsy in 411 consecutive patients with breast cancer.

## 2. Results and Discussion

### 2.1. Patients and Tumor Characteristics

The study group consisted of 411 patients from Kyoto University Hospital (Kyoto, Japan), Shinko Hospital, and Kobe City Central Hospital (Kobe, Japan). All patients were diagnosed by either fine-needle aspiration or core needle biopsy. Informed consent was obtained from the patients for lymphatic mapping and SLN biopsy using ICG and a blue dye as a prelude to SLNB. Their median age was 57.8 years (range, 30-91 years). Overall, 13.1% of patients (54 of 411) had DCIS, 82.5% (339 of 411) had invasive ductal carcinoma (IDC), and 12 patients (2.9%) with T3 tumors were assigned for SLN biopsy. The clinicopathological characteristics of the patients are summarized in Table 1.

**Table 1:** Patients and tumor characteristics

		No.	%
Age (years)	>50	131	31.9
	<50	280	68.1
Site	Area A	52	12.6
	Area B	15	3.7
	Area C	136	33.1
	Area D	36	8.8
	Area E	6	1.5
	Two areas	149	36.2
	More than two areas	17	4.1
Tumor size	Tis	54	13.1
	T1	215	52.3
	T2	112	27.3
	T3	12	2.9
	Missed	18	4.4
Histology	DCIS	54	13.1
	IDC	357	86.9
Grade	0	26	6.3
	1	153	37.2
	2	73	17.8
	3	79	19.2
	Missing	80	19.5
ER	Negative	104	25.3
	Positive	169	41.1
	Missing	138	33.6
PR	Negative	104	25.3
	Positive	169	41.1
	Missing	138	33.6
HER2/neu	Negative	212	51.6
	IHC2+	13	3.2
	IHC3+	28	6.35
	Missing	158	38.4

### 2.2. The Number of SLNs Removed and the Percentage of SLNs Involved

Nine hundred and fifty SLNs were removed and assessed using standard operative techniques. The mean number of SLNs removed per patient was 2.3 ± 1.2 (range, 1–9). The mean number of SLNs removed per patient at Kyoto University Hospital, Shinko Hospital and Kobe City Medical Center Central Hospital was 3.5 ± 1.7 (range, 1–9), 1.7 ± 0.79 (range, 1–4), and 2.3 ± 1.08 (range, 1–5), respectively (Table 2). At least one SLN was identified and removed in 408 patients, and the identification rate was 99%. Only one SLN was harvested in 30.1% of patients, two in 29.4%, three in 23.9%, and four or more in 15.9% of patients.

**Table 2:** The number of SLNs removed at the three institutes.

No. of SLNs removed	Kyoto University Hospital	Shinko Hospital	Kobe City Medical Center	Total (%)
1	7	36	8 84484	127 (30.1)
2	6	26	89	121 (29.4)
3	4	13	81	98 (23.9)
4	12	1	30	43 (11)
5	8	0	7	15 (3.7)
6	3	0	0	3 (0.7)
9	1	0	0	1 (0.2)
Total	41	76	291	408

Using this method, 99% (408/411) of SLNs were fluorescence positive. Detection rate of blue dye in these three institutes ranged from 83% to 93%. SLN involvement was found in 39 patients (9.5%) and the following ALNDs were completed. All SLNs containing tumor cells were fluorescence positive and 30 of 39 patients (77%) had one SLN involved (Table 3).

**Table 3:** The number of SLNs involved.

No. of SLNs involved	No. of patients	%
0	369	90.5
1	30	7.3
2	4	1
3	5	1.2
Total	408	100

At Kyoto University, 13 patients with positive SLNs underwent subsequent completeALND. Involvement of fluorescent SLNs was observed in all patients with positive lymph nodes. In these patients, 27 lymph nodes were harvested, and the ratio of metastatic SLNs to metastatic lymph nodes was 20/27 (74%).

### 2.3. Discussion

Sentinel lymph node biopsy is now widely accepted as an alternative to ALND, and a patient without SLN involvement can avoid standard axillary clearance in order to reduce morbidity factors associated with ALND, such as lymph edema or uncomfortable arm pain [5,6,7]. Regarding the agent used for detecting sentinel lymph nodes, technetium-99 (99mTc) sulfur colloids and 99mTc albumin colloids are commonly used worldwide. These radiocolloids are trapped in the SLNs and detected by a gamma probe. The radioisotope and blue dye compensate for each other and reduce the false negative rate [8]. This method does not require any special training, but only requires a facility equipped to deal with radioisotopes.

The ICG florescence method, a modification of the dye method, enables a surgeon to navigate to the axillary lymphatic basin along the subcutaneous lymphatic vessels. Near-infrared radiation of wavelength 765 nm activates ICG molecules and fluorescent emissions at a wavelength of 830 nm are detected on a PED system [3]. SLNs are identified as fluorescence positive and/or dye positive. The rate of fluorescence negative and dye positive results is no more than 5% (data not shown). This method enables orderly and sequential dissection along the lymphatic flow. In this study, data from 411 patients with breast cancer were analyzed. Subcutaneous lymphatic channels were visualized in all cases. The identification rate of SLNs was 99% (408/411), which is comparable to the rate achieved with the combined method using the radioisotope and dye [9,10,11,12]. At Kyoto University, Shinko Hospital, and Kobe City Medical Center, the detection rate using the blue dye method was 87%, 93%, and 92%, respectively. However, the overall detection rate was not calculated because many records did not classify SLNs harvested in terms of fluorescence and dye. Tagaya *et al*. [4] reported the use of ICG fluorescence imaging to detect SLNs after simultaneously injecting ICG (1 mL) and blue dye (3 mL of indigo carmine). They successfully identified SLNs in all 25 patients (100%) with fluorescence imaging and 19 of 25 patients with the blue dye staining method (92%). These results indicated that the detection rate using the blue dye was comparable with our results.

The radioisotope method enables navigation to the site of skin incision by measurement of radiation whereas the ICG fluorescence method can precisely identify the site of skin incision by tracing the lymphatic vessels across the skin. The confirmation of SLN removal using the radioisotope tracer is facilitated when less than 10% are left in the operation field after the first node is removed [13]. The fluorescence method, however, provides actual information regarding which lymph nodes should be removed as SLNs and whether an SLN biopsy was completed. In this study, a mean of 2.3 SLNs were removed per patient, which is a larger number than is the case with the radioisotope method. Hojyo *et al*. [14] recently reported that the average number of SLNs detected by fluorescence, dye and RI was 3.8, 1.9 and 2.0, respectively. As the ICG fluorescence technique can identify the basin that includes not only SLNs but also para-SLNs where the lymphatic vessels drain, the average number of lymph nodes removed also tends to increase. From the technical point-of-view, ICG leaks from damaged lymphatic vessels and this causes non-specific staining.

In the preclinical experiment, florescence signals were detected most efficiently at concentrations between 0.5–1 μg/mL in the test tube. The intensity of fluorescence signals tended to decrease at values below and above this range of concentration. Considering the amount of body fluid, we believe that giving 5 mg of ICG is reasonable in terms of osmotic and hydrostatic pressures. Murawa *et al*. [15] reported that the visualization of lymphatic flow was dose-dependent. Furthermore, they reported that subcutaneous lymphatic flow was identified most efficiently when ICG was administered at a concentration of 15 mg; however, the detection rate of SLNs was identical when both 5 mg and 10 mg of ICG was administered. They altered the administration volume and not the of ICG concentration, and hence, hydrostatic pressure may have contributed to the high identification rate of lymphatic flow. In our experience, 0.25 mg/mL of ICG is sufficient to detect SLNs and subcutaneous lymphatic flow. In conclusion, administering 1 mL of 5 mg/mL of ICG is currently common but the optimal dose of ICG is unclear. It is dependent upon the administration volume and concentration of ICG, so it should be assessed in future clinical trials.

The ICG technique achieves a high identification rate comparable to that of the radioactive method. This non-isotope method is simple and provides precise information on the lymphatic network of the breast. Orderly and sequential dissection along the lymphatic flow may provide higher sensitivity than the radioisotope method. SLN biopsy alongthe lymphatic flow leads to sequential dissection of SLNs according to anatomy whereas the quantification of ICG gives the functional aspect of SLNs. Quantification may provide complimentary information to confirm the order of SLN removal.However, there is no evidence as to whether the “hottest” node in the radioisotope method is identical to a maximally fluorescent node in the ICG method. The ICG fluorescence method cannot be confirmed as a substitute for the radioisotope method until a direct comparison between the methods is completed.

## 3. Experimental Section

### 3.1. Patients

In this retrospective study, the medical records of 520 patients with breast cancer who underwent successful SLN biopsy at Kyoto University Hospital (Kyoto, Japan), Shinko Hospital and Kobe City Medical Center Central Hospital (Kobe, Japan) were reviewed. The inclusion criteria were operable breast cancer at clinical stage cT1–3, N0, M0 and eligibility for SLN biopsy followed by breast conservation surgery or mastectomy. 

All patients were diagnosed either by fine needle aspiration or core needle biopsy. Exclusion criteria were clinically node-positive breast cancer, pregnancy and previous breast and/or axillary surgery. Only 411 of 520 patients had sufficient clinical data for analysis in this study.

### 3.2. ICG Fluorescence Technique

**Figure 1:**
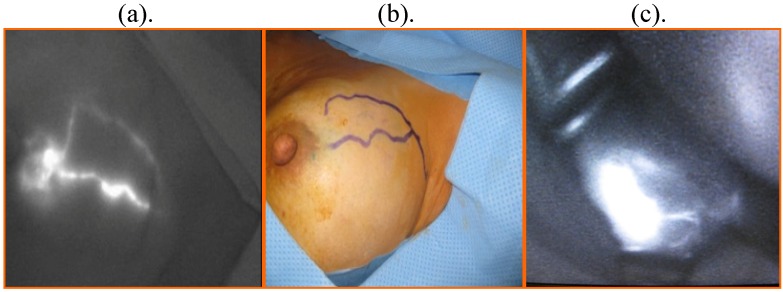
The ICG fluorescence technique for SLN biopsy (a) Subcutaneous lymphatic streams were visualized as fluorescent signals on PDE; (b) could be traced on the surface of the skin; and (c) a skin incision was made and the fluorescent basin was shown on the monitor.

ICG-guided near-infrared fluorescence imaging combined with a blue dye was used for SLN biopsy in all patients. After induction of general anesthesia, 1 mL of blue dye was injected into the periareolar area. Then, 1 mL of 0.5% ICG was injected into the periareolar area after sterilization of the operative site. Within a few seconds after injection, the fluorescent lymphatic stream could be detected on the skin surface by directing the photo dynamic eye (PDE) onto the breast. The fluorescent stream of the lymphatic flow in the subcutaneous lymphatic channels could be observed by PDE in real time and ICG uptake could be enhanced by massaging the injection point (Figure 1a).

The subcutaneous lymphatic channels were mapped over the skin until the point at which the fluorescent line disappeared near the axilla (Figure 1b). At this point, the lymphatic channels drained from the subcutaneous tissue into the axillary space. A skin incision of about 2 cm was made at the point where the lymphatic channels drain into the axilla. The subcutaneous connective tissue was dissected and PDE was reapplied to the incision area and the fluorescent basin areas including the sentinel lymph nodes were shown on the monitor (Figure 1c).

**Figure 2:**
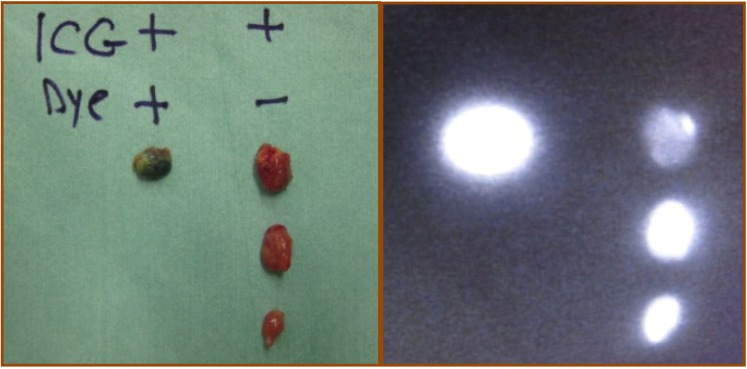
All removed lymph nodes were classified in terms of fluorescent signal and/or blue color.

The SLNs with or without blue dye lymph nodes were removed under the guidance of the monitor. PDE was further used to confirm whether the removed nodes were fluorescence positive or not. After removal, it was necessarily to ensure that no fluorescent spots were left in the axilla. All removed lymph nodes were classified according to their color and fluorescent signal as dye and ICG positive (double positive) or ICG positive (single positive) (Figure 2). Each group was examined separately by the pathologist for metastasis.

## 4. Conclusions

This ICG fluorescence method is simple and achieves a high SLN identification rate. This technique does not require a facility equipped to use radioisotopes. This means that SLN biopsies could even be performed in a small hospital. Orderly and sequential dissection along the lymphatic flow may provide higher sensitivity compared with the conventional radioisotope method. A direct comparison between the radioisotope and ICG fluorescence methods is now required.
